# Modulation of the lytic apparatus by the FtsEX complex within the bacterial division machinery

**DOI:** 10.1002/1873-3468.14953

**Published:** 2024-06-07

**Authors:** Martín Alcorlo, Siseth Martínez‐Caballero, Jianwei Li, Lok‐To Sham, Min Luo, Juan A. Hermoso

**Affiliations:** ^1^ Department of Crystallography and Structural Biology Instituto de Química‐Física “Blas Cabrera”, Consejo Superior de Investigaciones Científicas Madrid Spain; ^2^ Department of Biological Sciences, Faculty of Science National University of Singapore Singapore; ^3^ Department of Biological Sciences, Center for Bioimaging Sciences National University of Singapore Singapore; ^4^ Infectious Diseases Translational Research Programme and Department of Microbiology and Immunology, Yong Loo Lin School of Medicine National University of Singapore Singapore

**Keywords:** 3D structure, ABC transporters, bacterial division, divisome, FtsE, FtsX, mechano‐transmission, PG hydrolase

## Abstract

The FtsEX membrane complex constitutes an essential component of the ABC transporter superfamily, widely distributed among bacterial species. It governs peptidoglycan degradation for cell division, acting as a signal transmitter rather than a substrate transporter. Through the ATPase activity of FtsE, it facilitates signal transmission from the cytosol across the membrane to the periplasm, activating associated peptidoglycan hydrolases. This review concentrates on the latest structural advancements elucidating the architecture of the FtsEX complex and its interplay with lytic enzymes or regulatory counterparts. The revealed three‐dimensional structures unveil a landscape wherein a precise array of intermolecular interactions, preserved across diverse bacterial species, afford meticulous spatial and temporal control over the cell division process.

## Graphical abstract

Conformational changes involved in the mechano‐transmission activation of the lytic machinery by FtsEX. This model is based on the Gram‐negative bacterium *Pseudomonas aeruginosa*, highlighting the EnvC protein that facilitates the activation of the lytic enzyme AmiB during cell division.

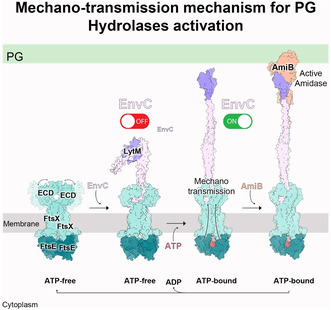

## Abbreviations

**ABC**, ATP‐binding cassette

**CC**, coiled‐coil

**CH**, coupling helix

**CryoEM**, cryo‐electron microscopy

**ECD**, extracellular domain

**ECL**, extracellular loop

**EH**, elbow helix

**Lip**, linear interacting peptide region

**MSP**, membrane scaffold protein

**NBD**, nucleotide‐binding domain

**PG**, peptidoglycan

**TM**, transmembrane segment

**TMD**, transmembrane domain

The peptidoglycan (PG) constitutes an intricate molecular scaffold enveloping the bacterial cytoplasmic membrane, imparting structural stability and shielding against osmotic pressure [1]. Comprising repeating units of N‐acetylglucosamine and N‐acetylmuramic acid, PG undergoes polymerization and cross‐linking *via* stem peptides attached to N‐acetylmuramic acid, resulting in the formation of a cell‐shaped sacculus enclosing the cytoplasmic membrane [2]. Throughout the cell cycle, PG undertakes substantial restructuring to facilitate the expansion and division of the cell envelope [3, 4]. In numerous bacterial species, enzymes involved in PG remodeling assemble into one of two supramolecular complexes: the Rod complex (or elongasome) and the divisome, which mediate PG synthesis during elongation and division, respectively. Divisome assembly commences with the formation of the Z‐ring, comprising dynamic polymers of FtsZ, which subsequently recruits numerous cell division proteins to the mid‐cell region. During Z‐ring formation, FtsA and ZipA, which are early divisome components, tether FtsZ to the cytoplasmic membrane at the prospective division site. This process is succeeded by the ordered assembly of several division proteins, including FtsEX, FtsQLB, FtsWI, and FtsN [5, 6]. Upon divisome maturation, it becomes activated to induce cell constriction and PG synthesis, reinforcing the daughter cell poles. The resulting septal PG, initially shared by the daughter cells, necessitates cleavage to complete division [7, 8, 9, 10].

Peptidoglycan remodeling is intricately linked to cytokinesis through FtsEX. Apart from its pivotal role in cell division, the FtsEX complex has been involved in several different cellular processes (recently reviewed in Ref. [11]). Within the cellular division machinery of Gram‐negative bacteria, FtsE engages with the C‐terminal tail of FtsZ [12], establishing a connection between the Z‐ring and the transmembrane component FtsX. This implies a potential role for FtsEX as a membrane fixative for FtsZ, employing ATP binding and hydrolysis to govern Z‐ring constriction [13, 14]. Furthermore, FtsEX plays a direct role in recruiting periplasmic division PG hydrolases to the division site.

It belongs to the type VII family of ATP‐binding cassette (ABC) transporters [15], encompassing members such as MacB [16], LolCDE [17, 18, 19], and HrtBA [20]. The core architecture of FtsEX consists of two copies of FtsE, acting as intracellular Nucleotide‐Binding Domains (NBDs), and two copies of FtsX. FtsX comprises one Transmembrane Domain (TMD) and an extracellular domain (ECD) responsible for binding to its cognate partner [15]. However, instead of transporting a substrate, the FtsEX complex propagates conformational changes across the cytoplasmic membrane [15, 21]. Essentially, FtsE interacts with FtsX, which is positioned within the membrane. The ATPase activity of cytoplasmic FtsE energizes the system by hydrolyzing ATP. Subsequently, the signal propagates from cytosolic FtsE through the TMDs of FtsX to its two ECDs through conformational changes [22, 23], and then, to the exposed coiled‐coil (CC) domain‐containing cognate binding partners. These CC domain proteins may act as additional regulators, facilitating interaction between FtsEX and hydrolases, such as the EnvC‐AmiB system in *Escherichia coli* [24]. Alternatively, the CC domain protein itself can function as the hydrolase, as illustrated by PcsB [25], CwlO [26, 27] or RipC [28, 29]. In *Corynebacterium*, RipA interacts with FtsEX and another activator, SteAB [27].

Very recently, the successful isolation and reconstitution of the full‐length FtsEX complexes from various organisms using peptidiscs or detergents, have facilitated their structural characterization by cryo‐electron microscopy (cryoEM). Peptidiscs represent a novel technology designed to stabilize membrane proteins in a detergent‐free environment [30, 31]. They consist of a self‐assembling amphipathic peptide, adapted from membrane scaffold proteins (MSPs), which forms a protective belt around the hydrophobic regions of membrane proteins, maintaining their solubility in aqueous solutions. Despite not requiring specific lipids during reconstitution, peptidiscs have demonstrated a comparable ability to preserve protein functionality similar to that of nanodiscs. The resulting structures, including those of *E. coli* (8W6J, 8W6I and 8HD0 [32]), *Pseudomonas aeruginosa* (8I6Q, 8I6O, 8I6R and 8I6S [23]), *Mycobacterium tuberculosis* (8IDB, 8JIA, 8IDC, 8IGQ, and 8IDD [33]) and *Vibrio cholerae* (8TZL and 8TZK [34]), revealed important information on FtsEX functioning and its role in regulation of PG hydrolases. In this review, we will systematically outline the main advances of this system, initiating with the cytosolic facet (FtsE), progressing through the cellular membrane (FtsX), and concluding with elements situated in the periplasmic space or external to the cell.

### FtsE serves as the cytosolic ATPase component energizing the FtsEX complex

**Fig. 1:**
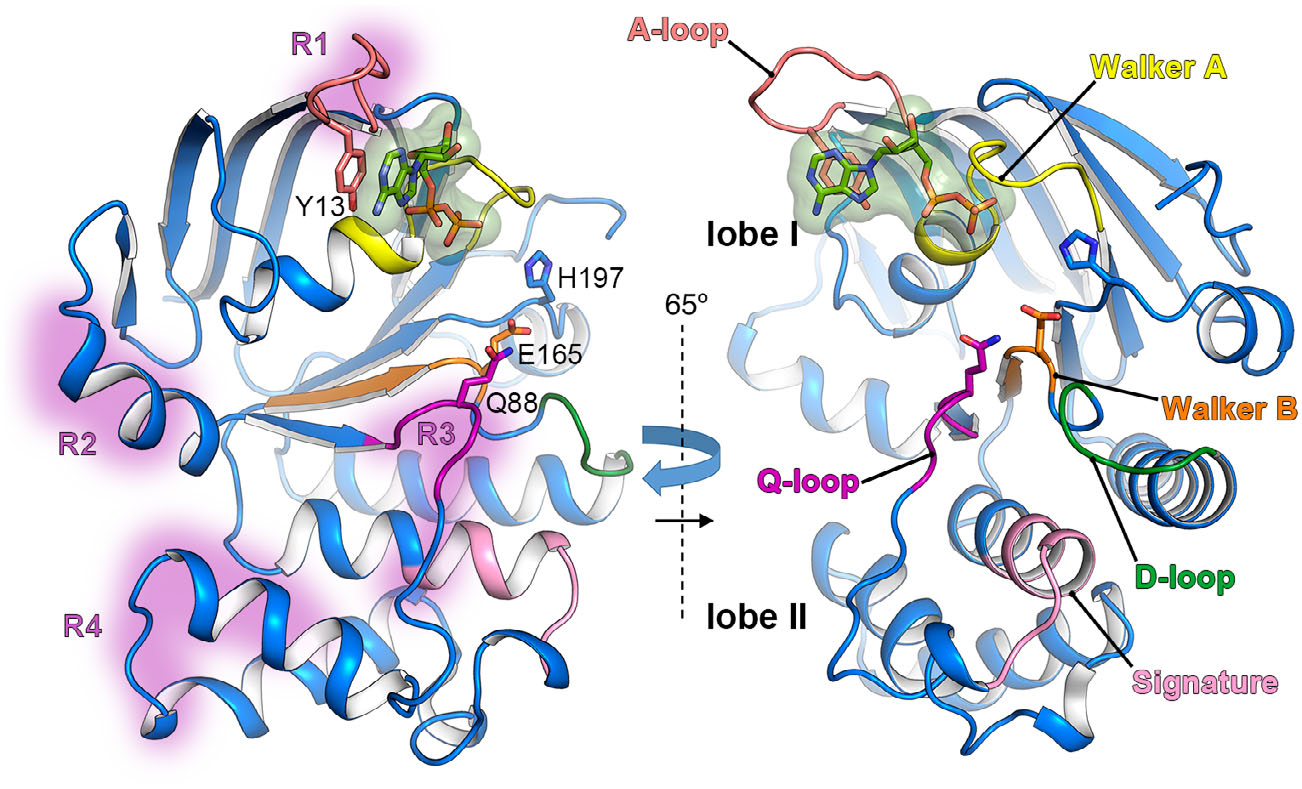
Crystal structure of FtsE from *Streptococcus pneumoniae* bound to ADP. Monomeric FtsE is depicted in a blue‐ribbon representation (PDB 6Z4W [36]), emphasizing conserved and functionally critical motifs, such as Walker A/B, A/D/Q‐loops and the signature motif (colored in yellow/orange, salmon/green/magenta and pink; respectively). The two views are related by a 65° turn along the indicated axes. ADP is represented in sticks with a semitransparent molecular surface. Specific FtsE residues, such as the glutamate involved in catalysis (E165), the conserved glutamine (Q88) from the Q‐loop, the aromatic side chain (Y13) from the A‐loop, and the switch histidine (H197), are depicted in capped sticks. Specific regions on the FtsE monomer (left panel) where structural plasticity is prominent are painted in pale purple (R1, R2, R3, and R4).

FtsE is one of the initial proteins recruited to the divisome and directly interacts with FtsZ [14]. In addition, very recently it was shown that FtsE plays a role in coordinating septal PG synthesis with bacterial cell division [35]. The FtsE structures reported up to date underscore a substantial conservation in the primary sequence (identity ranging 42–64%) and a highly conserved 3D structure (*rmsd* values ranging 0.87–1.47 Å, upon structural superposition), emphasizing its pivotal role in cell division. As observed in the high‐resolution crystal structures of FtsE of *Streptococcus pneumoniae* (6Z67, 6Z63, and 6Z4W [36]), FtsE comprises two distinct thick lobes (Fig. 1), labeled as lobe I and II, with conserved motifs and critical functional regions inherent to NBDs associated with ATPase activity in the ABC transporter family [37, 38]. These features encompass (a) the Walker A motif (also termed phosphate‐binding loop or P‐loop), facilitating nucleotide binding; (b) the Walker B motif with a Mg ion‐binding site and housing the glutamate that participates in catalysis; (c) the A‐loop, contributing an aromatic side chain for purine ring packing; (d) the switch region, containing a stabilizing histidine residue during ATP hydrolysis; (e) the α‐helical subdomain featuring the signature motif (LSGGE), a characteristic of the ABC transporter superfamily, guiding ATP orientation during hydrolysis; (f) the Q‐loop (presenting a conserved glutamine), positioned between the signature and Walker A motifs, interacting with the γ‐phosphate and linking to the TMD; and (g) the dimerization or D‐loop, facilitating dimerization and coupling hydrolysis to transport. The nucleotide‐binding site comprises the Walker A/B, the switch region, and the A/Q‐loops [39, 40, 41]. *In vivo*, FtsE demonstrates catalytic activity as a dimer, where each FtsE monomer positions its conserved motifs towards the other monomer. This specific arrangement leads to the creation of two ATP‐binding sites positioned between the P‐loop and the signature motif, with each motif originating from a different monomer [36].

The lobe I of FtsE is characterized by a mixture of α‐ and β‐structures, housing the Walker A/B motifs, alongside essential residues crucial for ATP hydrolysis and/or binding located within the P‐loop. The ATP‐binding cavity is situated at the periphery of lobe I. Lobe II, orthogonal to lobe I, is exclusively α‐helical and incorporates the signature motif. The hinge region connecting lobe I and lobe II encompasses both the switch region and the Q‐loop, establishing contact with the ATP γ‐phosphate.

The structural analysis of pneumococcal FtsE [36] also unveiled a pronounced conformational plasticity concentrated in four distinct regions (R1–R4). Among these, three regions (R2–R4) collectively form a hydrophobic cavity that accommodates the cytosolic segment of FtsX, specifically the coupling helix (CH, see below). These identified regions were postulated to exert a pivotal role in influencing the transmission of signals from the cytosol through the membrane, thereby regulating lytic activity within the periplasmic space [36].

### FtsX serves as the membrane component within the FtsEX complex

**Fig. 2:**
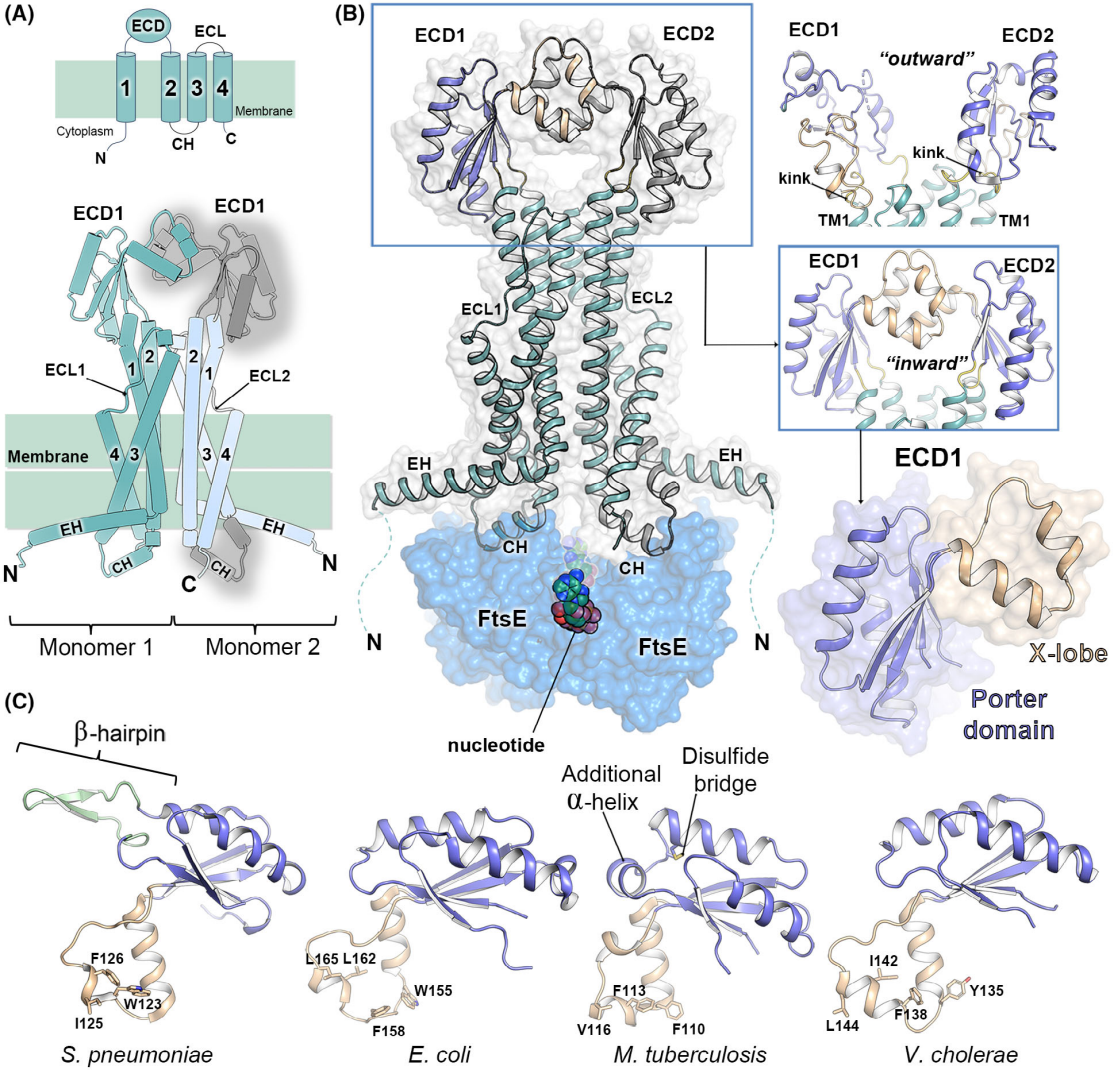
Three‐dimensional structure of the dimeric FtsX. (A) Diagram cartoon illustrating the topology of the FtsX monomer (upper panel) and dimer (lower panel). TM regions are numbered from 1 to 4. In the lower panel, one FtsX monomer is fully colored in green, while the other monomer displays TMs colored in cyan. The ECDs from each monomer are labeled, with ECD from monomer 2 (ECD2) colored in gray along with the CH. (B) Left panel, cartoon depiction of the *Pae*FtsX structure in complex with FtsE (PDB 8I6R [23]). The FtsX molecular surface is semitransparent and colored gray, while the FtsE dimer is represented as a semitransparent blue surface. Nucleotide molecules are depicted as spheres. The orientation of FtsX is consistent with lower panel A. Likers connecting the ECDs to TM1 and TM2 are colored yellow. In ECD1, the upper lobe (X‐lobe) is colored purple, and the lower lobe (Porter Domain) is depicted in wheat. The same color code applies for the right lower panel. ECD2 is fully colored in gray. The right upper panel illustrates the ECDs from *Mtb*FtsEX (PDB 8IDB [33]), with the two lower lobes oriented in opposite directions (referred to as the “outward” conformation). The right middle panel mirrors the representation of the ECDs in the right panel of B, showcasing their orientation with the lower lobes directed towards each other (termed the “inward” conformation). (C) Comparison of FtsX_ECD_ domains depicted in cartoon format. The Porter domain is purple, and the X‐lobe is wheat‐colored. Structural data originates from PDB entries 6HFX (*Streptococcus pneumoniae* [45]), 6TPI (*Escherichia coli* [46]), 4N8N (*Mycobacterium tuberculosis* [29]) and 8TZL (*Vibrio cholerae* [34]). Specific structural features of each ECD are labeled, while pivotal hydrophobic residues within the X‐lobe, crucial for the interaction with the CC of their respective binding partners, are enumerated and represented in stick form. C, C‐terminus; CH, coupling helix; ECL, large extracellular loop; EH, elbow helix; N, N‐terminus. See main text for details.

As very recently discovered through the structural characterization of the full‐length protein [23, 33], FtsX emerges as an integral membrane protein featuring four transmembrane segments (TM1–TM4), with both the amino and carboxyl termini placed at the cytosol (Fig. 2A). The primary dimerization interface of FtsX involves TM1 and TM2 [23, 33]. TM1 adopts an L‐shape, housing an amphipathic elbow helix (EH) running parallel to the membrane. While TM1, TM2, and TM3 protrude outside the membrane, TM4 is a short helix completely embedded within it. TM1 and TM2 connect to an approximately 100‐residue‐long folded ECD. The ECD comprises the so‐called Porter subdomain and the X‐lobe (Fig. 2B). Lastly, TM3 and TM4 are connected through a small extracellular loop (ECL). These regions (ECD and ECL) participate in interactions with either cell wall hydrolases or effector proteins, ultimately triggering the activation of PG cleavage. [22, 24, 25, 27, 29, 42, 43]. Importantly, the dimeric ECDs create a groove accessible for partner protein binding (see below).

Contrary to the observed sequence conservation in FtsE, the sequence identity in FtsX is notably lower (from 17% to 39%). Interestingly, the CH of FtsX (Fig. 2B) located between TM2 and TM3 that interacts with FtsE, exhibits a significant degree of sequence conservation. As mentioned above, the CH is inserted in FtsE, in the hydrophobic cavity shaped by regions R2‐R4 [36].

The N‐terminus of FtsX (encompassing residues 53–70 in *E. coli*), which includes the EH, contains ~ 50 additional residues in Gram‐negative bacteria (such as *E. coli* and *P. aeruginosa*) that are nonexistent in Gram‐positive organisms. In the case of *E. coli*, this region has been shown to interact with FtsA [12, 44], thus linking the FtsEX complex with the components of the cell division ring.

Several high‐resolution ECD structures have been documented for various bacteria including *M. tuberculosis* (4N8N [29]), *S. pneumoniae* (e.g., 6HFX [45]) and *E. coli* (6TPI [46]) (Fig. 2C). Despite originating from different bacteria, these structures share a common architecture characterized by a bilobed arrangement. Within the upper lobe, a central core is discernible, composed of two βαβ secondary structure motifs intricately interlocked to form a central four‐membered β‐sheet flanked by two helices. Remarkably, this domain bears resemblance to the Porter subdomain identified in *E. coli* MacB and LolC, a conserved feature found across all type VII ABC transporters and integral to the mechano‐transmission apparatus [16, 17] (see below). The lower lobe, referred to as the X‐lobe [15], consists mainly of α‐helices and encompasses a conserved α‐helix ~ 10‐residues in length, alongside a shorter α‐helical region that exhibits potential for adopting different conformations, which reflects its dynamic behavior and conformational plasticity. Noteworthy, structural disparities among the available ECD structures include the presence of an elongated β‐hairpin in pneumococcus (a characteristic feature of streptococcal species [45]) and the occurrence of a disulfide bridge within the *M. tuberculosis* ECD (C73‐C78), which substitutes the β‐hairpin insertion observed in the pneumococcal ECD. In addition, the *M. tuberculosis* ECD features an additional short α‐helix at the Porter domain (residues 64–68) absent in the other FtsX ECDs. Notably, the X‐lobe, rigorously well‐preserved in FtsX, is nonexistent in other type VII ABC transporters, emphasizing its significance for FtsX‐specific functionalities. Across all documented 3D structures of the ECD, the upper and lower lobes demarcate a substantial hydrophobic crevice, previously identified as a primary candidate for the interaction interface between FtsX and its specific PG hydrolase or regulator [29]. These structures consistently exhibit hydrophobic residues, predominantly Phe and Trp residues, (Fig. 2C), situated in the distal portion of the X‐lobe, crucial for engaging with the binding partner (see below). A notable conformational difference arises when contrasting the dimeric configuration of *Mtb*FtsEX with other Type VII ABC transporters. Particularly, while the dimeric ECD of other Type VII ABC transporters, such as MacB [16], LolC [47] and the recently resolved *Pae*FtsEX [23] or *Eco*FtsEX [32], exhibit an orientation where their lower lobes are directed towards each other (“inward” conformation), the two ECD domains of *Mtb*FtsEX are positioned in opposite directions, facing outward (“outward” conformation).

This structural distinction is attributed to the presence of an evident kink observed in TM1 of *Mtb*FtsEX, a feature nonexistent in *Pae*FtsEX, *Eco*FtsEX, and other Type VII structures. This kink, occurring at the hinge region of TM1 of *Mtb*FtsEX, establishes a direct connection to the ECD, inducing a modification in its orientation and resulting in a unique conformation wherein the two ECD hooks align with their lower lobes facing in opposite directions. These lower lobes play a crucial role in binding cognate partners as will be discussed later on.

### Structure of PG hydrolases EnvC, RipC and PcsB

**Fig. 3:**
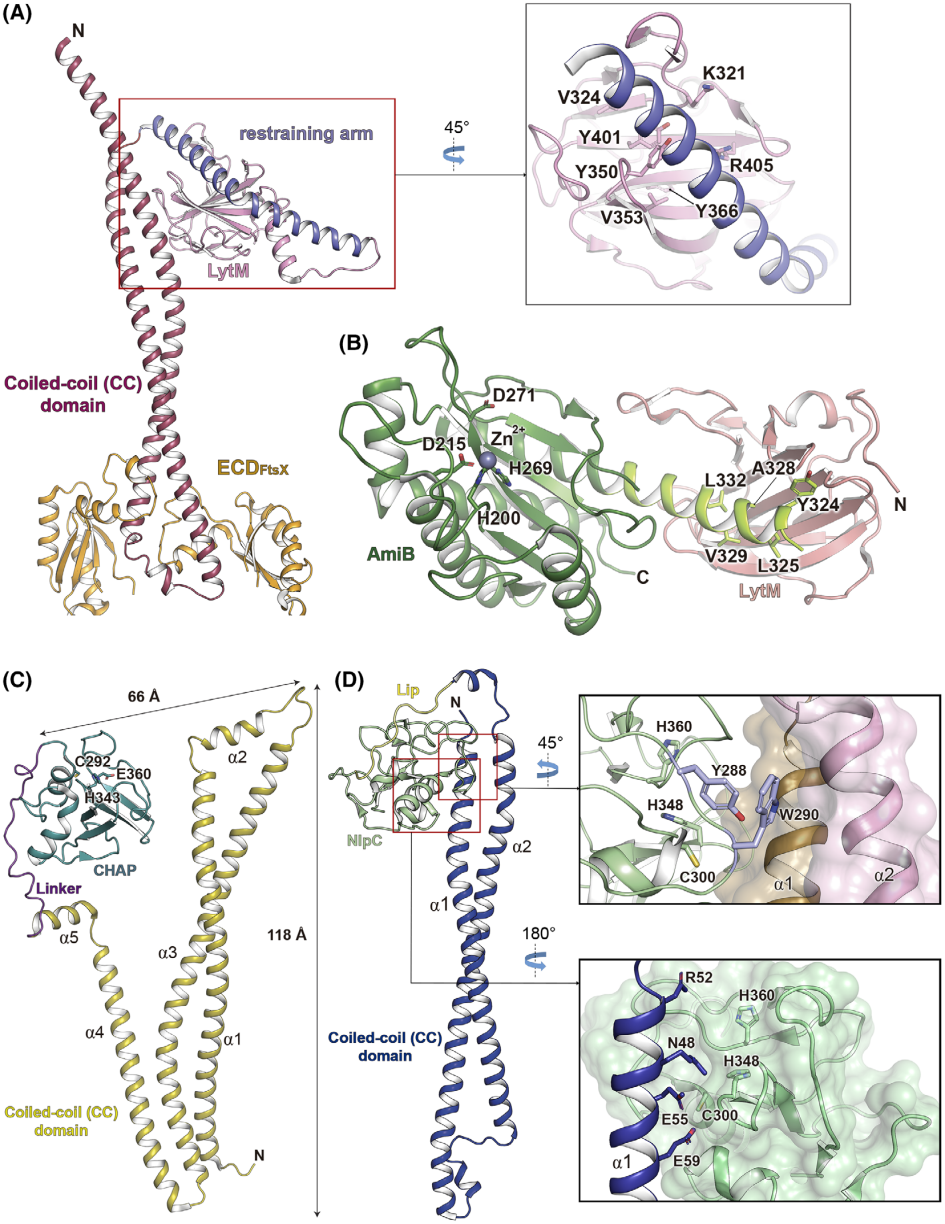
Three‐dimensional structures of EnvC, PcsB, and RipC. (A) Crystal structure of EnvC bound to FtsX_ECD_ (PDB 4TPI [46]). The inset presents a magnified view, schematically representing the amino acids of the LytM domain (depicted as sticks) engaged in interaction with the restraining arm. (B) Crystal structure of the AmiB domain (colored in green) bound to the cognate LytM domain (colored in pink) from *Citrobacter rodentium* (PDB 8C0J [52]). The α3 helix of AmiB, highlighted in light green, is nestled within the LytM groove of EnvC. Catalytic residues and those involved in the interaction with the LytM domain are depicted as sticks. (C) Crystal structure of PcsB (PDB 4CGK [42]), with catalytic residues of the CHAP domain depicted as sticks. (D) Structure of RipC (PDB 8IDC [33]). The upper right panel presents a schematic representation of amino acids Y288 and W290 (depicted as violet sticks) of the Nlp60 domain inserted between the α1 and α2 helices of the CC domain. The α1 helix is depicted as a brown surface, the α2 helix as a pink surface, and the catalytic domain as a green cartoon. The lower right panel illustrates residues of the CC situated in the α1 helix (depicted as blue sticks) interacting with the Nlp60 domain, shown as green surface. Catalytic residues of the Nlp60 domain are depicted as green sticks in both right panels.

In Gram‐negative bacteria like *E. coli*, the FtsEX system interacts with EnvC. This protein acts solely as a regulator for the activation of amidases AmiA and AmiB [48], which play pivotal roles in catalyzing hydrolytic reactions that facilitate cell separation [49]. The crystallographic structure of EnvC bound to FtsX_ECD_ was determined (PDB 6TPI [46]) with a stoichiometry of 2 : 1 (ECD_FtsX_ : EnvC). The full‐length EnvC structure comprises three domains: a CC domain situated in the N‐terminus (residues 35–216), encompassing residues 114–160 which engage with the two ECDs of FtsX; a regulatory segment termed “restraining arm” and a globular LytM domain situated in the C‐terminus (Fig. 3A) [46, 50]. The N‐terminal region of the CC (residues 35–216) comprises two extensive antiparallel helices (α1 and α2). This domain is distinguished by the presence of 21 heptad repeats, which are conserved among EnvC homologs. The α1 comprises 12 heptad repeats, while the α2 comprises 9 heptad repeats. Analysis of these heptad repeats reveals a predominance of hydrophobic residues such as leucine, isoleucine or valine in the first position, with leucine or glutamine being the most frequent at the fourth and fifth positions. Interactions between heptads from α1 and α2 facilitate the assembly of the CC. Intriguingly, heptad 22 is disrupted, forming a linker that connects the CC to the restraining arm. The restraining arm encompasses three additional disrupted heptad repeats (heptads 23–25), situated within the groove of the LytM domain (Fig. 3A) [46].

The LytM domain of EnvC is classified as a member of the zinc metallopeptidases M23 family (IPR016047) [50]. PG hydrolases belonging to this family feature three conserved amino acids (two His and one Glu) responsible for coordinating a catalytic Zn^2+^ cation within the active site, essential for their hydrolytic activity. However, the LytM domain of EnvC exhibits a degenerated active site cleft lacking residues involved in coordinating zinc (W320, V324, and Y401), which are typically conserved in other LytM domains. This deficiency accounts for its absence of hydrolase activity (PDB 4BH5 [50]). *Via* genetic selection and site‐directed mutagenesis, specific residues within the LytM pocket responsible for the activation of *E. coli* amidases have been identified both *in vivo* and *in vitro* [50]. This degenerated LytM domain has recently been observed in DipM as well [51], a regulator of cell division in *Caulobacter crescentus*. DipM interacts with various autolysins, including the soluble lytic transglycosylases SdpA and SdpB, along with the amidase AmiC. Mutations within this groove effectively disrupt DipM's function *in vivo* and its interaction with AmiC and SdpA *in vitro* [51].

Recently, the crystal structure of AmiB bound to the LytM domain of EnvC from the Gram‐negative bacteria *Citrobacter rodentium* was reported (PDB 8C0J [52]). This structure revealed a significant movement of the regulatory helix of AmiB, which shields the enzyme's active site, leading to enzyme activation (Fig. 3B). Moreover, the structure of AmiA from *E. coli* was resolved (PDB 8C2O [52]), revealing a comparable inhibitory mechanism involving a regulatory α3 helix, consistent with previous findings regarding AmiB [53].

In *S. pneumoniae*, akin to other Gram‐positive bacteria, FtsEX establishes direct interactions with its corresponding PG hydrolase. Notably, PcsB serves as the indispensable hydrolase essential for pneumococcal cell division. The crystal structure of PcsB (PDB 4CGK [42]) revealed the presence of a CC domain (residues 41–266), a linker region (residues 267–288), and a globular catalytic CHAP domain (residues 279–392) (IPR007921) [42]. The N‐terminal CC domain comprises five alpha helices, encompassing three elongated (α1, α2, and α4) and two short helices (α3 and α5) (Fig. 3C). Hydrogen bonds, leucine zipper motifs, and salt links predominantly stabilize the helices within the CC domain. Acting as a bridge between the CC and the CHAP domain, the linker presents an alanine‐rich sequence (266‐AAAPVRAKVRP‐279). The CHAP domain, located in the C‐terminus, adopts a classical papain‐amidase fold, housing the conserved catalytic residue C292 situated within the active site cleft (Fig. 3C). Through structural and sequence analyses, it was proposed that a catalytic triad (comprising residues C292, H343 and E360) operates within this domain. This hypothesis is reinforced by previous findings where point mutations of C292A and H342A were found to be non‐viable in pneumococcus [54]. Furthermore, the conserved positions of this catalytic triad are mirrored in the secreted CHAP domain from *Staphylococcus saprophyticus* [55]. The overall architecture of PcsB exhibits a characteristic V‐shape, characterized by two elongated sides measuring ~ 112 and 118 Å, and a shorter side spanning ~ 66 Å (Fig. 3A). The lack of *in vitro* activity in PcsB [42, 56, 57, 58] is explained by an autoinhibition mechanism, given that murein hydrolase activity has been exclusively demonstrated for the CHAP domain [42]. This implies that the muralytic activity of PcsB could be modulated by its CC domain. Notably, the observed PcsB structure in the protein crystal reveals a dimeric self‐inhibited configuration, which was confirmed at high protein concentrations in solution using small‐angle X‐ray scattering and analytical ultracentrifugation experiments. Conversely, at lower protein concentrations, a self‐inhibited monomeric form predominated [42]. Within the PcsB autoinhibition model, the α4 helix may serve a similar function to the restraining arm observed in EnvC (Fig. 3A) [32, 46], requiring a conformational change to release the CHAP domain and initiate activation [11].

In a manner reminiscent of Gram‐positive bacteria, the PG amidase RipC of *M. tuberculosis* directly interacts with its cognate partner FtsEX. Notably, the CryoEM structure of FtsEX in complex with RipC has recently been elucidated (PDB 8IDC [33]). The RipC structure unveils a unique arrangement, featuring a CC domain at the N‐terminal region (residues 45–216), a 52 amino acid‐long proline‐rich linker (residues 217–269), and a catalytic domain belonging to the NlpC/P60 family (IPR000064) at the C‐terminal region (residues 270–385). The CC domain comprises two elongated helices: α1 (residues 45–110) and α2 (residues 128–127), connected by an 18‐residues segment (residues 110–128) known as the Linear interacting peptide region (Lip) (Fig. 3D) [33].

The catalytic domain of RipC, akin to other members of its protein family, harbors a conserved catalytic triad comprising residues C300, H348, and H360. Analogous to the findings reported for the CHAP domain of PcsB [42], the catalytic domain of RipC adopts an autoinhibited state. This inactive conformation is maintained by two aromatic amino acids (Y288 and W290) from the catalytic domain, which insert into a groove situated between the α1 and α2 helices of the CC domain (Fig. 3D). Additionally, interactions with the N‐terminal region of α1 (residues 47–62) contribute to maintaining this state. Moreover, the catalytic residues are hindered by four polar residues (N48, R2, E55, and E59) *via* hydrogen bonds (Fig. 3D). Despite the completion of a model facilitated by the CryoEM‐derived map, density was observed only for the first 12 amino acids (residues 217–228) of the linker connecting the CC and catalytic domain. Remarkably, this portion of the linker exhibited significant stabilization through hydrophobic interactions with a large and deep groove (approximately 31 Å × 12 Å × 8 Å) of the NlpC/P60 domain located on the opposing side of the catalytic site [33]. Moreover, within the same investigation, it was noted that ATP hydrolysis by FtsE might be essential to initiate the activation of the RipC hydrolase (see below).

### Molecular bases of the interaction of FtsX with EnvC and RipC

**Fig. 4:**
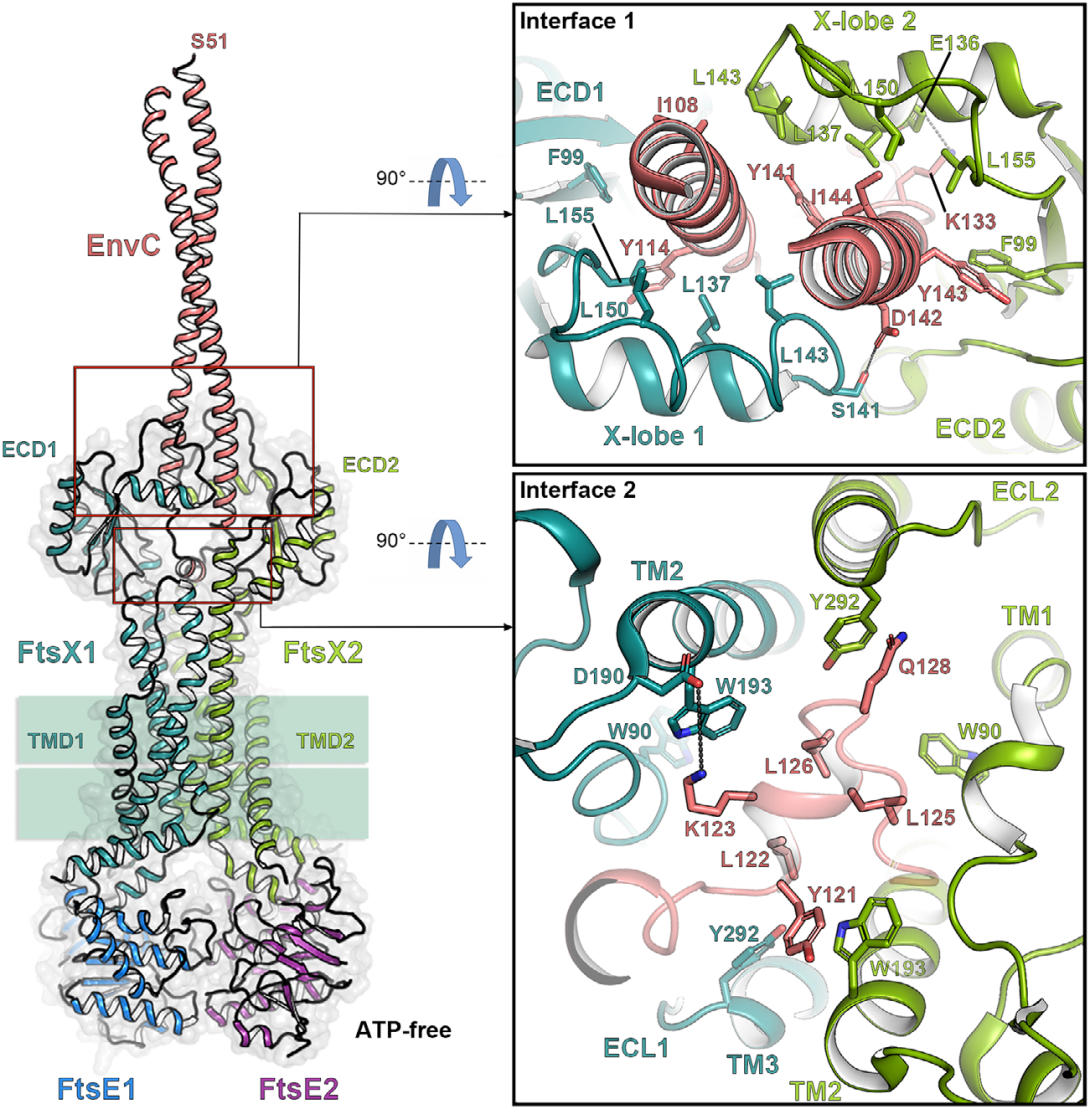
Recognition of EnvC by FtsX in *Pseudomonas aeruginosa* (ATP‐free state). Left panel, overall cartoon structure representation of the FtsEX–EnvC complex (PDB 8I6O [23]). FtsE monomers are colored in blue and purple, FtsX monomers are colored in light green and dark green, and bound EnvC is colored in salmon. Right upper panel, detailed interactions at interface 1 between FtsX and EnvC (Top‐Down view). Right lower panel, detailed interactions at interface 2 between FtsX and EnvC (Top‐Down view). Interface 1 involves extensive hydrophobic contacts between residues F99, L137, L143, L150, and L155 of the X‐lobe and residues I108, Y114, Y140, Y141, Y143, and I144 of EnvC. Interface 2, located at the hinge region of the EnvC CC domain, TM1, TM2, TM3, and the ECL of FtsX, also features interactions. Residues Y121, L122, L125, L126, and Q128 of EnvC interact with W90, W193, and Y292 of FtsX. Additionally, a salt bridge (EnvC/K123‐FtsX/D190) likely stabilizes the complex through electrostatic interactions. Polar interactions are depicted through dotted black lines. Residues involved are shown in capped sticks.

It has been recently investigated how EnvC (*E. coli*, *P. aeruginosa,* and *V. cholerae*) or RipC (*M. tuberculosis*) activities are modulated by the FtsEX system and the mechanism by which it triggers the corresponding PG hydrolase [23, 32, 33, 34]. The overall arrangement of the FtsEX:EnvC complex is similar in *E. coli* and *P. aeruginosa*, where the CC domain of EnvC intercalates from above between the two ECDs along the central axis of FtsEX. We will detail the case of *Pae*FtsEX complex which interacts with EnvC in a 2 : 2 : 1 stoichiometry [23]. One end of the EnvC CC domain is inserted between the two ECDs of FtsX. The interaction between EnvC and FtsX is formed by two interfaces (Fig. 4). The first interface involves the CC region of EnvC (residues 103–117, residues 135–152) and hydrophobic residues of the X‐lobe (Fig. 4). The second interface, also hydrophobic, is situated at the hinge region of the EnvC CC domain, the TM1‐3 and the ECL of FtsX [23].

In *P. aeruginosa*, several loops of FtsX (particularly the X‐lobe and the ECL) are pivotal in facilitating interactions with EnvC. The inherent flexibility of these loops, coupled with the hydrophobic interactions they mediate, endows FtsEX with the adaptability required for binding to the asymmetrical EnvC (Fig. 4). It is worth mentioning that in the absence of ATP, the cryoEM structure of *Pae*FtsEX:EnvC revealed solely the presence of the CC domain of EnvC (Fig. 4). It was anticipated that EnvC would adopt an inactive conformation under these conditions, wherein the lytM domain is blocked by one helix of the CC domain. This observation aligns with the findings from the crystallographic analysis of the ECD:EnvC complex in *E. coli* [46].

**Fig. 5:**
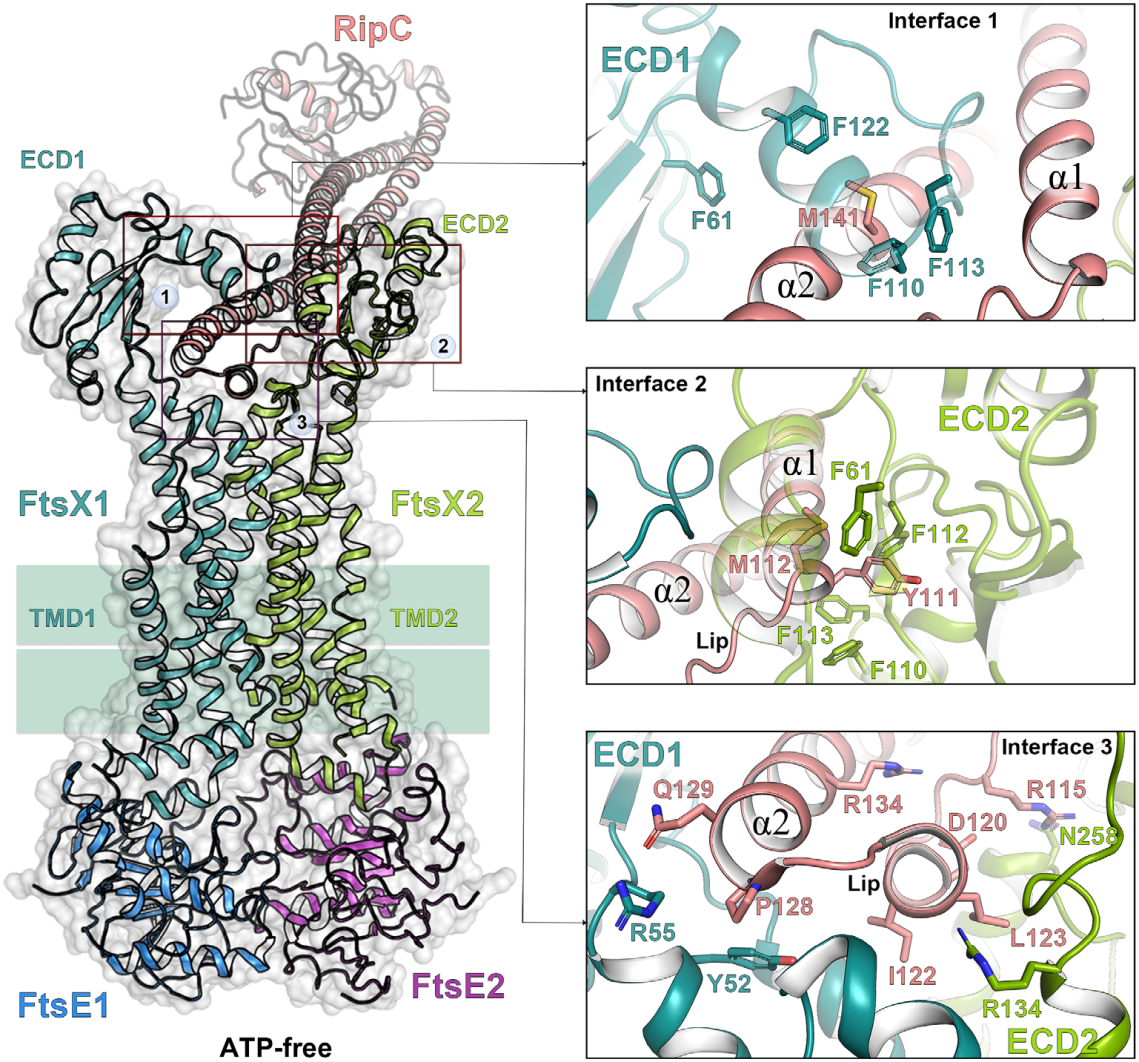
Recognition of RipC by FtsX in *Mycobacterium tuberculosis* (ATP‐free state). Left panel, overall cartoon structure representation of the FtsEX–RipC complex (PDB 8IDC [33]). FtsE monomers are depicted in blue and purple, FtsX monomers in light green and dark green and bound RipC is colored in salmon. Right upper, middle, and lower panels provide detailed insights into the interactions between FtsX and RipC at interfaces 1, 2, and 3; respectively. Critical residues involved in recognition are depicted in capped sticks. Interfaces 1 and 2 feature a Phe cluster comprising four phenylalanine residues (F61, F110, F113, and F122) from each of the two lower lobes of the ECDs. These Phe residues are closely packed against RipC, with Y111 on the complementary side of α1_RipC_ engaging in multiple π–π interactions with the Phe cluster; while its neighboring M112 forms a robust methylene‐aromatic interaction with F61. Additionally, M141 from α2_RipC_ participates in a methylene‐aromatic interaction with the Phe cluster from another ECD. Interface 3 comprises the top surface area of the TM helices with the Lip portion of RipC and predominantly consists of hydrophobic interactions, including residues Y52 or I122. Furthermore, a few hydrogen bonds, such as R115_RipC_‐N258_FtsX_ and Q129_RipC_‐R55_FtsX_, are also observed.

In *M. tuberculosis*, similarly to the systems described above, the interaction between RipC and the ECDs of FtsX entails specific regions of RipC binding directly to FtsX (Fig. 5). The overall binding pocket for RipC resembles a bone joint cavity, effectively encapsulating the bulky region stemming from the Lip side of RipC, thereby facilitating the protrusion of the distal end of RipC's NlpC/P60 domain into the extracellular space [33]. This interaction is not directly affected by ATP hydrolysis and is characterized by three major interfaces driven by extensive hydrophobic interactions. Briefly, a Phe cluster located in the ECD, in conjunction with hydrophobic residues, plays a crucial role in complex formation (see legend of Fig. 5 for details) [33].

Remarkably, the FtsEX:RipC complex (PDB 8IDC [33]) exhibits notable distinctions compared to the EnvC‐bound *Pae*FtsEX (PDB 8I6O [23]). While both interactions occur between the two ECDs, EnvC's CC aligns closely with the central axis of FtsEX, yielding an elongated conformation (Fig. 4). Conversely, RipC inserts into the ECD domain from the side at an approximate ~ 116° tilt from the central axis, resulting in an inclined binding mode (Fig. 5). In the *Mtb*FtsEX complex with RipC, one ECD monomer aligns with the central axis, owing to a straight hinge conformation from TM1 and TM2. However, the other ECD monomer exhibits a kink in the hinge of TM1, causing the lower lobe of the ECD to diverge from the central axis at an angle of ~ 90°. Consequently, while EnvC in *Pae*FtsEX passes between the two parallel ECD hooks, RipC in *Mtb*FtsEX can only bind from the side and is restricted by the lower lobe of the ECD hook oriented towards the central axis.

Hence, while in Pseudomonas both ECDs undergo symmetrical movements to trap the EnvC partner, in Mycobacterium, the repositioning of both ECDs is asymmetric. While one ECD retains a disposition akin to the apo conformation, the other undergoes significant conformational changes. This movement likely results from the partial refolding of residues 49–55, causing the large kink to fold into an α‐helix upon RipC recognition. As a result, this enlarges TM1 and facilitates a straightened conformation for the kink [33].

### Coupling ATPase activity to mechano‐transmission

**Fig. 6:**
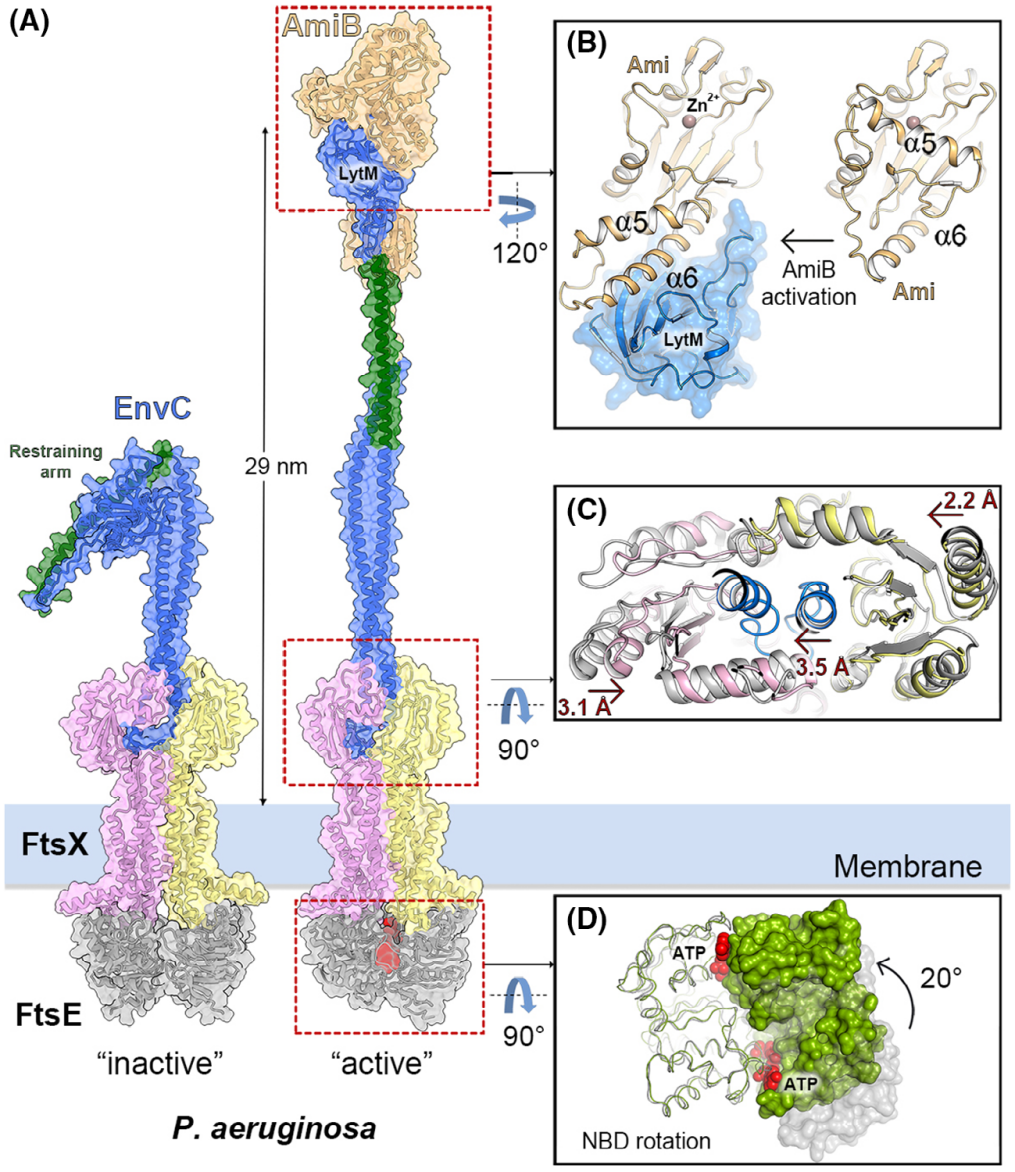
Mechano‐transmission activation of AmiB in the *Pae*FtsEX:EnvC system. (A) Activation of AmiB. Left panel; AlphaFold prediction of the autoinhibited EnvC–FtsEX complex in *Pseudomonas aeruginosa* with EnvC shown in blue and its restraining arm in green. One FtsX monomer is pink and the other yellow, while both cytoplasmic FtsE monomers are gray. The AmiB amidase is depicted in orange. All structures are presented in cartoons with semitransparent surfaces. Right panel; relocation of the restraining arm (green) and LytM domain upon activation within the FtsEX–EnvC–AmiB supercomplex (PDB 8I6S [23]). (B) AmiB activation. The relocation of α6 from the catalytic domain enables the liberation of the catalytic site of AmiB for PG hydrolysis. Right panel; in the structure of the catalytic domain of AmiB (Ami), α5 is shown obstructing the catalytic site. Left panel; in the activated supercomplex, as a consequence of the interaction between α6 and the LytM domain, there is a relocation of α5 that exposes the catalytic site. The catalytic Zinc ion is depicted as a brown sphere. (C) Comparison with the structure of ATP‐free *Pae*FtsEX/EnvC. The ATP‐free structure is shaded in gray (PDB 8I6O [23]). In the presence of ATP, the ECDs undergo movements ~ 3.1 and 2.2 Å from two sides, causing a compression of one of the helices from the CC domain of EnvC by 3.5 Å. (D) ATP binding induces a 20° rotation of one FtsE monomer (colored in green). ATP is represented as red spheres. One FtsE monomer is depicted in ribbon whereas the other is displayed as a molecular surface. The ATP‐free structure of FtsE is depicted in gray (PDB 8I6O [23]). The ATP‐bound FtsE structure is colored green (PDB 8I6S [23]). These structural alterations initiate subsequent conformational changes in EnvC and AmiB.

Peptidoglycan hydrolases controlled by FtsEX are autoinhibited, regardless of their reliance on an activator such as EnvC. This autoinhibition, when the PG hydrolase is an amidase, is mediated by a regulatory helix that occludes the active site of the hydrolase. This helix can be displaced through an “inside‐out” mechano‐transmission mechanism (Fig. 6B). For instance, in the *Pae*FtsEX:EnvC:AmiB system, ATP binding induces a rotation of 20° of one of the FtsE subunits (Fig. 6D), leading to inward movement of the two ECDs of FtsEX by ~ 3 and ~ 2 Å, from each side, resulting in the compression of the CC domain of EnvC by ~ 3.5 Å (Fig. 6C). The conformational changes propagated through the CC domain of EnvC extend to the periplasmic end of the helix, releasing the LytM domain and repositioning the regulatory helix (“restraining arm”) of EnvC (Fig. 6A). The LytM domain subsequently binds to the ⍺6 helix of AmiB, causing the α5 helix to move, unblocking the active site and triggering PG hydrolysis. The elongated CC domain of EnvC also positions the AmiB molecule to cleave the connecting PG ~ 29 nm away from the inner membrane (Fig. 6A,B) [23].

**Fig. 7:**
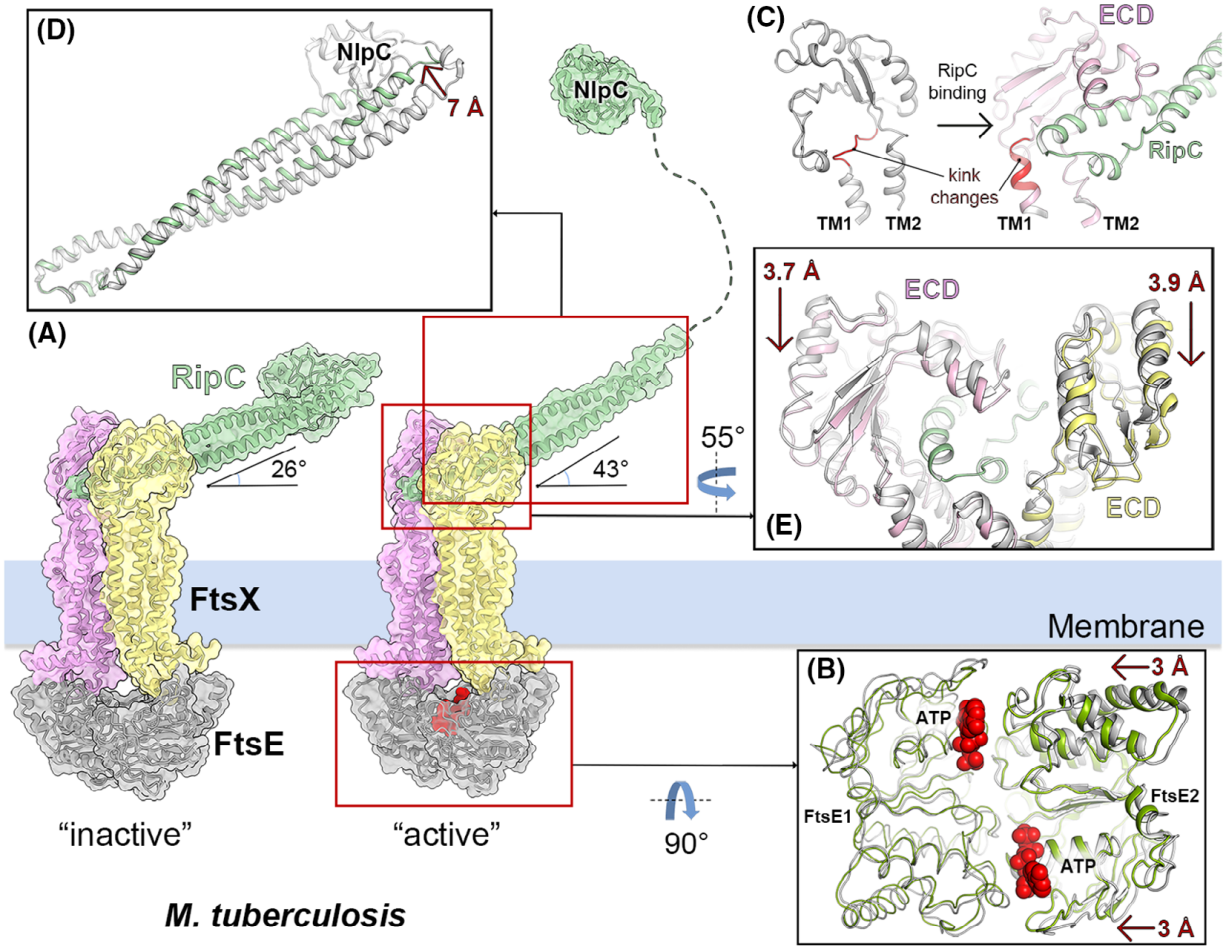
Mechano‐transmission activation of RipC in the *Mtb*FtsEX:RipC system. (A) Left panel; autoinhibited RipC–FtsEX complex (ATP‐free) in *Mycobacterium tuberculosis* with RipC shown in green (PDB 8IDC [33]). One FtsX monomer is pink and the other yellow, while both cytoplasmic FtsE monomers are gray. All structures are presented in cartoon form with semitransparent surfaces. Right panel; conformational changes in the FtsEX:RipC complex upon activation after ATP binding (PDB 8IDD [33]). In the activated form the NlpC catalytic domain is not visible due to its mobility. To illustrate the release of the NlpC domain upon activation, it is depicted linked to the complex through dashed lines. (B) Conformational changes of FtsE upon ATP binding. ATP is represented as red spheres. One FtsE monomer is depicted in ribbon whereas the other is displayed in cartoon. The FtsE structure without ATP is depicted in gray (PDB 8IDC [33]), while the ATP‐bound FtsE structure is colored green (PDB 8IDD [33]). (C) Switch between a straight (pink) and a kinked conformation (gray) of the hinge region of the TM1 governs the orientation of the linked ECD monomer for RipC binding, with the kink region in red and bound RipC in green. (D) Conformational changes in RipC during the transition from ATP‐free state to ATP‐bound state. (E) Conformational alterations in the ECDs are observed during the transition from ATP‐free state to the ATP‐bound state. In panels B, C, D, and E; the ATP‐free structures (PDB 8IDC [33]) are colored in gray (see main text for further details).

The mechano‐transmission mechanism of the FtsEX:RipC system in *M. tuberculosis* is similar (Fig. 7A). ATP binding to the FtsE subunits induces a 3.5 Å shift in one of the NBDs (Fig. 7B). This shift moves the two FtsE molecules closer and initiates the conformation changes in FtsX, particularly at the hinge region connecting two transmembrane helices, TM1 and TM2, to the ECD. This region is kinked and changes its conformation upon RipC binding (Fig. 7C). Although this conformational alteration is relatively subtle, it results in a compression of the interacting interface of bound RipC by ~ 3.8 Å, causing the CC domain of RipC to ascend by ~ 7 Å (Fig. 7D) [33]. Subsequent changes in the conformation of FtsEX lead to an inward movement of the ECD by an additional ~ 3 Å (Fig. 7E). The mechanical signal transmitted through the CC domain ultimately liberates the catalytic NlpC/p60 domain of RipC to initiate PG cleave (Fig. 7A, right panel). This signal may also elongate the proline‐rich linker of RipC, allowing the NlpC/p60 domain to access the ~ 14 nm space between the inner membrane and the PG layer (Fig. 7) [33].

Insights into the structural rearrangement following ATP hydrolysis may be gleaned from a recent ADP‐bound structure of EnvC‐FtsEX in *V. cholerae* [34]. Instead of adopting the ‘vertically extended’ conformation observed in the ATP‐bound state, the ADP‐bound EnvC‐FtsEX complex adopts a ‘horizontally extended’ configuration, similar to the inclined RipC‐FtsEX complex of *M. tuberculosis*. The tilted CC domain of EnvC likely relocates the dLytM domain and AmiB away from PG, thereby spatially modulating its hydrolytic activity. The subsequent release of ADP restores the complex to its resting state by rotating the CC domain back to the vertically extended conformation, preparing it for another cycle of activation.

## Concluding remarks

**Fig. 8:**
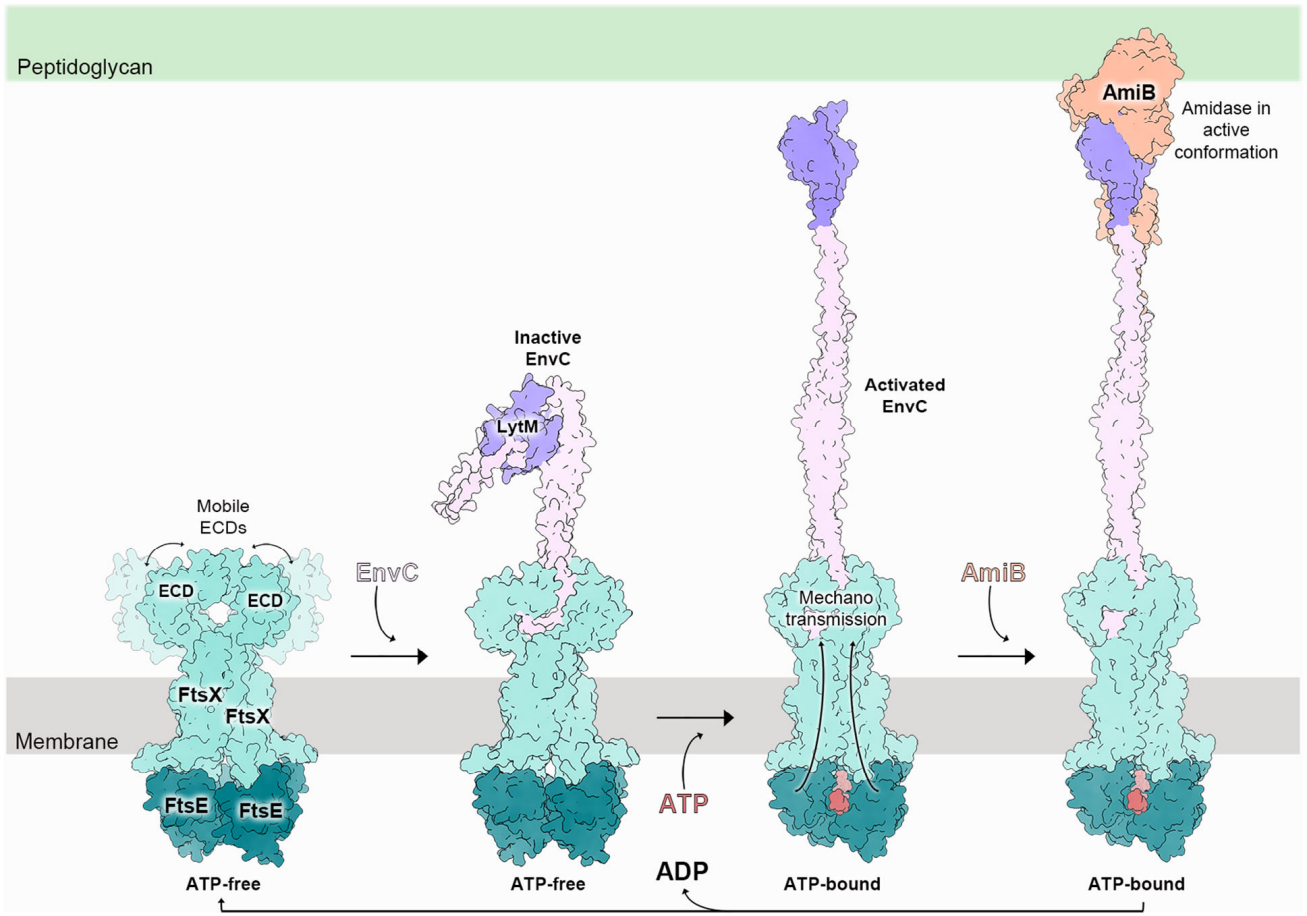
Scheme illustrating the events involved in the FtsEX mechano‐transmission activation of lytic machinery. The scheme is based on the Gram‐negative *Pseudomonas aeruginosa* system, presenting the EnvC protein that mediates the activation of the lytic enzyme AmiB.

A recent structural analysis of PG activation across various bacteria, including *S. pneumoniae*, *P. aeruginosa*, *E. coli*, *V. cholerae,* and *M. tuberculosis*, reveals a relatively conserved “inside‐out” mechano‐transmission mechanism (Fig. 8). Initially, in the ATP‐free resting state, the two NBD domains are spatially separated in the cytosol. In this state, the cognate partners situated outside the membrane, such as EnvC and the autoinhibited hydrolase/amidase, maintain their original inactive state. Upon ATP binding/hydrolysis, NBDs engagement at the cytosolic side induces minor movements of TM1 and TM2, embedded within the membrane. This force is then transmitted through the hinge regions to the ECDs, resulting in the compression of the two ECDs outside the membrane. Subsequently, this compressive effect propagates to the CC domains of either EnvC or the directly bound PG hydrolase, causing the release of their restraining arm and subsequent activation. Thus, a sequential cascade of conformational changes transmits signals from the cytosolic FtsE across the membrane through the TM helices of FtsX, and ultimately to the ECDs, which interact with EnvC or PG hydrolases, activating these cognate binding partners. Activation of these partners leads to activation of the associated amidase in Gram‐negative bacteria, as observed in the cryoEM structure of *Pae*FtsEX.EnvC:AmiB complex [23], in the crystal structures of *Ccr*AmiB:LytM [52], or in the liberation of the catalytic domain of the PG hydrolase as occurs in *M. tuberculosis* [33] or as also must occur in Gram‐positive bacteria [42], ultimately activating the PG cleavage.

Moreover, in addition to activating autoinhibited EnvC and hydrolase/amidase domains for PG cleavage activity, an additional layer of mechano‐transmission orchestrates the precise positioning of these domains for targeted PG cleavage. This regulatory mechanism involves modulating the location of the catalytic site of hydrolases/amidases, primarily through two distinct mechanisms: tilting the CC domain, as exemplified in the RipC system in *M. tuberculosis*, or elongating the CC domain, as observed in both *P. aeruginosa* and *E. coli* systems.

The positioning of the hydrolase/amidase catalytic site outside the membrane appears to be linked to two major cell division types; (a) in *P. aeruginosa* and *E. coli* FtsEX/EnvC systems, the vertical binding mode of EnvC and elongation of the CC domain enable the activated AmiB to reach beyond (~ 30 nm away) the typical distance of the PG layer from the membrane (< 20 nm). This spatial arrangement allows the activated amidase active site to selectively cleave the connecting layers of the septal PG while leaving stress‐bearing PG untouched. In contrast, (b) in the FtsEX/RipC system in *M. tuberculosis*, the inclined binding mode and absence of a mediator like EnvC restrict the activated RipC's reach to a shorter distance from the membrane (about 8–14 nm), facilitating cleavage of stress‐bearing PG closer to the membrane. These structural insights from different systems provide partial explanations for the two major observed cell division types: one involving septal PG cleavage in most Gram‐negative bacteria and the other selectively targeting stress‐bearing PG. This selective targeting potentially alters the mechanical properties of the cell envelope, facilitating a “V‐snapping” mode of cell division in Corynebacterineae.

Future structural work will provide further insights into the regulatory details controlled by the FtsEX system as well as other potential partners that could contribute to this pivotal process.
